# Machine Learning Approaches to Predict Symptoms in People With Cancer: Systematic Review

**DOI:** 10.2196/52322

**Published:** 2024-03-19

**Authors:** Nahid Zeinali, Nayung Youn, Alaa Albashayreh, Weiguo Fan, Stéphanie Gilbertson White

**Affiliations:** 1 Department of Computer Science and Informatics University of Iowa Iowa City, IA United States; 2 College of Nursing University of Iowa Iowa City, IA United States; 3 Department of Business Analytics University of Iowa Iowa City, IA United States

**Keywords:** machine learning, ML, deep learning, DL, cancer symptoms, prediction model

## Abstract

**Background:**

People with cancer frequently experience severe and distressing symptoms associated with cancer and its treatments. Predicting symptoms in patients with cancer continues to be a significant challenge for both clinicians and researchers. The rapid evolution of machine learning (ML) highlights the need for a current systematic review to improve cancer symptom prediction.

**Objective:**

This systematic review aims to synthesize the literature that has used ML algorithms to predict the development of cancer symptoms and to identify the predictors of these symptoms. This is essential for integrating new developments and identifying gaps in existing literature.

**Methods:**

We conducted this systematic review in accordance with the PRISMA (Preferred Reporting Items for Systematic Reviews and Meta-Analyses) checklist. We conducted a systematic search of CINAHL, Embase, and PubMed for English records published from 1984 to August 11, 2023, using the following search terms: *cancer*, *neoplasm*, *specific symptoms*, *neural networks*, *machine learning*, *specific algorithm names*, and *deep learning*. All records that met the eligibility criteria were individually reviewed by 2 coauthors, and key findings were extracted and synthesized. We focused on studies using ML algorithms to predict cancer symptoms, excluding nonhuman research, technical reports, reviews, book chapters, conference proceedings, and inaccessible full texts.

**Results:**

A total of 42 studies were included, the majority of which were published after 2017. Most studies were conducted in North America (18/42, 43%) and Asia (16/42, 38%). The sample sizes in most studies (27/42, 64%) typically ranged from 100 to 1000 participants. The most prevalent category of algorithms was supervised ML, accounting for 39 (93%) of the 42 studies. Each of the methods—deep learning, ensemble classifiers, and unsupervised ML—constituted 3 (3%) of the 42 studies. The ML algorithms with the best performance were logistic regression (9/42, 17%), random forest (7/42, 13%), artificial neural networks (5/42, 9%), and decision trees (5/42, 9%). The most commonly included primary cancer sites were the head and neck (9/42, 22%) and breast (8/42, 19%), with 17 (41%) of the 42 studies not specifying the site. The most frequently studied symptoms were xerostomia (9/42, 14%), depression (8/42, 13%), pain (8/42, 13%), and fatigue (6/42, 10%). The significant predictors were age, gender, treatment type, treatment number, cancer site, cancer stage, chemotherapy, radiotherapy, chronic diseases, comorbidities, physical factors, and psychological factors.

**Conclusions:**

This review outlines the algorithms used for predicting symptoms in individuals with cancer. Given the diversity of symptoms people with cancer experience, analytic approaches that can handle complex and nonlinear relationships are critical. This knowledge can pave the way for crafting algorithms tailored to a specific symptom. In addition, to improve prediction precision, future research should compare cutting-edge ML strategies such as deep learning and ensemble methods with traditional statistical models.

## Introduction

### Background

Cancer poses considerable physical and psychological challenges for those diagnosed with the disease. The Global Cancer Observatory estimated that there were 19.3 million new cancer cases and 43.8 million individuals living with cancer within 5 years of diagnosis globally in 2020 [1]. Symptoms such as fatigue, pain, nausea, vomiting, depression, and anxiety often persist beyond treatment [2-5], detrimentally affecting individuals’ quality of life [6]. Moreover, people with cancer frequently grapple with multiple intertwined symptoms [7], intensifying their distress [8]. Unmanaged cancer symptoms can lead to increased health care use, including emergency department visits and unscheduled hospitalizations to address these symptoms; a decline in the quality of life [9]; and even a reduced life expectancy. Providing precision symptom management tailored to the individual at the right moment has the potential to significantly improve outcomes, which is crucial for both people with cancer and their health care providers. Accurately predicting and addressing these symptoms is fundamental to providing such precision in symptom management.

Artificial intelligence, incorporating machine learning (ML) and deep learning (DL) models, excels in handling complex, high-dimensional, and noisy data. It has demonstrated effectiveness in disease diagnosis, predicting disease recurrence, enhancing quality of life, and symptom management [10-16]. There is a growing interest in ML in the emerging field of predictive analytics for cancer symptoms. ML contributes to the development of robust clinical decision systems, enhancing overall health care delivery [17]. ML algorithms can be broadly categorized into supervised learning, unsupervised learning, semisupervised learning, and reinforcement learning. DL, a subset of ML, addresses complex tasks such as speech recognition, image identification, and natural language processing [18].

### Objectives

This study seeks to offer a comprehensive and systematic review of the literature on the application of ML algorithms in predicting symptoms for people with cancer. Conducting this review of a rapidly expanding body of literature is imperative to understand the current state of the science for ML models in symptom prediction for cancer and to guide future research. This research aims to provide a comprehensive understanding of the current state of research; identify areas for improvement; and understand the limitations and gaps in the current literature, such as a lack of specific focus on ML models for patients with cancer. By comparing model performances across diverse symptom prediction tasks, we can identify the best practices, highlight areas for improvement, and offer informed recommendations that will propel the field of predictive analytics in cancer symptom research forward.

## Methods

### Search Strategy and Data Sources

This study was conducted in accordance with the PRISMA (Preferred Reporting Items for Systematic Review and Meta-Analyses) protocol [19] and involved a comprehensive database search spanning from 1984 to August 11, 2023, including the PubMed, Embase, CINAHL, and Google Scholar databases. The search terms encompassed *cancer*, *neoplasm*, *signs and symptoms*, *neural networks*, *machine learning*, and *specific algorithm names*. In our study, we used Boolean expressions, using specific combinations of keywords and phrases, acknowledging the variability in terminology across studies. Search results were compiled using EndNote 20 (Clarivate Analytics). The detailed search strategy, which uses Boolean expressions, and the PRISMA checklist can be found in Multimedia Appendices 1 and 2.

### Inclusion and Exclusion Criteria

To identify relevant research focusing on the application of ML methods in predicting cancer symptoms, we applied the following inclusion criteria: (1) papers published in English, (2) studies that used ML algorithms, and (3) research specifically aimed at predicting cancer symptoms. The exclusion criteria were as follows: (1) nonhuman studies, (2) technical reports, (3) review papers, (4) book chapters or series, (5) conference proceedings, and (6) studies for which full texts were unavailable. Two authors, NZ and NY, independently screened and cross-checked the candidate records. During the screening process, conducted using EndNote 20, any disagreements were resolved by consulting a third reviewer (SGW). The screening process involved an initial review of titles and abstracts, followed by a full-text examination to determine the study’s eligibility for inclusion in the review.

### Data Extraction and Analysis

In our study, we implemented a systematic, multistep process for data synthesis. Initially, relevant studies were identified and selected based on the predefined inclusion and exclusion criteria. Two independent researchers, NZ and NY, extracted data from 42 selected studies. They worked independently to mitigate bias and enhance the accuracy of the data extraction process. In cases of discrepancies, these were resolved through discussion or consultation with a third reviewer, SGW. The extracted data were aggregated, involving the collation of study characteristics such as research location, sample size, study design, types of ML algorithms, validation metrics, identified significant predictors, cancer types, and the specific symptoms focused on. This comprehensive approach enabled us to reduce the bias and increase the reliability of our findings. For the analysis, we used both quantitative and qualitative methods. Quantitative data, such as frequencies and percentages, were compiled and analyzed using Python. This included the creation of insightful plots and heat maps to identify patterns and trends, illustrating relationships among variables and highlighting key findings in an easily digestible format. Qualitative aspects, such as algorithm implementation or study design, were explored through narrative synthesis. This allowed for a deeper understanding of the context and nuances in the application of ML algorithms for cancer symptom prediction.

We conducted a cross-analysis to compare findings from different studies, assessing the effectiveness of various ML algorithms across different cancer types and symptoms and identifying common predictors of success and the challenges faced. Finally, we interpreted the findings in the context of the existing literature. We discussed how our results align with or differ from previous studies and what new insights our synthesis brings to the field of ML in cancer symptom prediction.

## Results

### Overall Results

**Figure 1:**
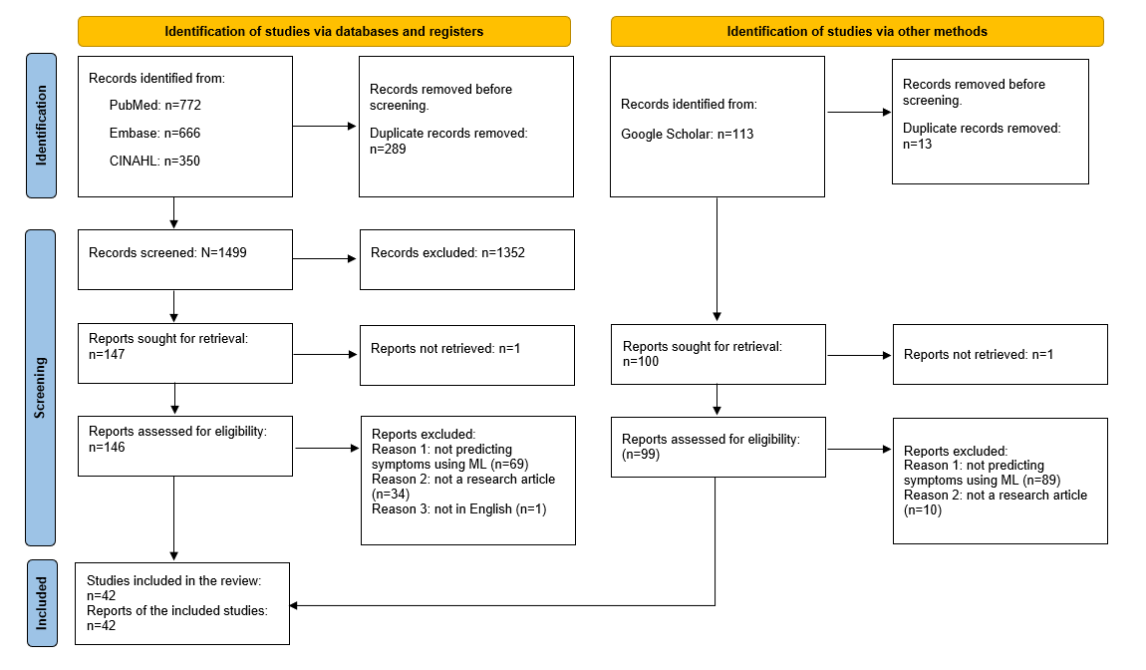
PRISMA (Preferred Reporting Items for Systematic Reviews and Meta-Analyses) flowchart. ML: machine learning.

A search across the 3 databases produced 1788 papers. After removing 289 duplicates, we screened the records for titles and abstracts, excluding another 1352 irrelevant records. However, 1 study was not retrieved. We reviewed the full text of the remaining 146 records, omitting 105 due to the absence of ML application in predicting cancer symptoms (69/146, 47.3%), not being a research article (34/146, 23.3%), and not being an English article (1/146, 1%). In the second phase, we intend to include Google Scholar in our research methodology to capture an additional 113 articles not found in our main databases, although 1 study was not retrieved. We reviewed the full text of the remaining 99 records, ultimately excluding all of them for reasons such as the lack of ML applications in cancer symptom prediction (89/99, 90%) and not being a research articles (10/99, 10%). Eventually, 42 studies met the inclusion criteria, as depicted in Figure 1.

Of the 42 studies, 42 (100%) is listed in PubMed, Embase covers 37 (88%) studies, and CINAHL includes 18 (43%) studies. The distribution and overlap of these research articles across the databases are illustrated in Multimedia Appendix 3.

The data extracted from these studies, which include the reference number, research location, year, data type, cancer site, symptoms, significant predictors, ML algorithms, and validation methods, are detailed in Table 1 and in Multimedia Appendix 4.

**Table 1:** Details of the included studies (n=42).

Study	Country, year	Data type; number of data	Population	Cancer symptoms	Significant predictors	Algorithms	Validation methods
Sun et al [20]	China, 2023	Clinical data; 1152	People with breast cancer	Pain	Postmenopausal status, urban medical insurance, history of at least 1one operation, underwent general anesthesia with fentanyl and sevoflurane, and received axillary lymph node dissection.	*LR^a,b^*, *RF^c^*, *GBDT^d^*, and *XGB^e^*	Random
Xinran et al [21]	China, 2023	Clinical data; 494	People with advanced cancer	Cognitive impairment	Cancer course, anxiety, and age	LR and *ANN^f^*	Random
Shaikh et al [22]	United States, 2023	Clinical data; 1152	Survivors of cancer with osteoarthritis	Depression	Age, education, care fragmentation, polypharmacy, and zip code–level poverty	*XGB*	10-fold CV^g^
Kober et al [23]	United States, 2023	Clinical data; 1217	People with cancer receiving chemotherapy	Morning fatigue	13 individual Li-Fraumeni syndrome items	*EN^h^*, RF, LASSO^i^, LR (filtered/unfiltered), RPAR^j^, and SVM^k^	Random
Du et al [24]	China, 2023	Clinical data; 565	People with cancer	Fatigue	Pain score, Eastern Cooperative Oncology Group score, platelet distribution width, and continuous erythropoiesis receptor activator	LR, *RF*, NB^l^, and XGB	5-fold CV
Moscato et al [25]	Italy, 2022	Clinical data; 21	People with cancer	Pain	N/A^m^	SVM, RF, *MP^n^*, LR, and AdaBoost^o^	10-fold CV
Masukawa et al [26]	Japan, 2022	Clinical data; 808	People with cancer	Social distress, spiritual pain, pain, dyspnea, nausea, and insomnia	N/A	*LR, RF, light GBM^p^, SVM,* and *ensemble*	5-fold CV
Fanizzi et al [27]	Italy, 2022	CT^q^ image data; 61	People with oropharyngeal cancer receiving radiotherapy	Xerostomia	Weight preradiotherapy, induction chemotherapy, sex, platinum-based chemotherapy, current chemotherapy, alcohol history, age at diagnosis, smoking history, surgery, clinical tumor, and clinical node	SVM and *CNN^r^*	10-fold CV
Ueno et al [28]	Japan, 2022	Clinical data; 284	People with breast cancer	Insomnia	General fatigue, physical fatigue, and cognitive fatigue	*L2 penalized LR* and *XGB*	8-fold CV
On et al [29]	Korea, 2022	Clinical data; 935	People with cancer receiving chemotherapy	Nausea-vomiting, fatigue-anorexia, diarrhea, hypersensitivity, stomatitis, hand-foot syndrome, peripheral neuropathy, and constipation	Earlier history of adverse drug reaction, comorbidity, cancer site and type of chemotherapy, demographics, and antineoplastic therapy–related features	*LR, DT^s^*, and *ANN*	3-fold CV
Li et al [30]	China, 2022	Clinical data and CT image data; 365	People with cancer receiving radiotherapy	Xerostomia	Hypertension, age, total radiotherapy dose, dose at 50% of the left parotid volume, mean dose to right parotid gland, mean dose to oral cavity, and course of induction chemotherapy	*RF*, DT and XGB	External validation
Kurisu et al [31]	Japan, 2022	Clinical data; 668	People with advanced cancer receiving pharmacological interventions	Delirium	The baseline Delirium Rating Scale-R98 severity score (cutoff of 15), hypoxia, and dehydration	*DT*	5-fold CV
Guo et al [32]	China, 2022	Clinical data; 80	People with lung cancer receiving chemotherapy	Lung infection	Age ≥60 years, length of stay ≥14 days, surgery history, combined chemotherapy, myelosuppression, diabetes, and hormone application	LR and *ANN*	Random
Baglione et al [33]	United States, 2022	Clinical data; 40	People with breast cancer	Depressed mood and anxiety	Connectedness, receive support, frequency and duration use of mobile app, and physical pain	*RF* and XGB	LOOCV^t^
Chao et al [34]	United States, 2022	Clinical data and CT image data; 155	People with HNC^u^ receiving radiotherapy	Xerostomia	N/A	SVM, *KNN^v^*, NB, and RF	Nested
Wakabayashi et al [35]	Japan, 2021	Clinical data and CT image data; 69	People with cancer receiving radiotherapy	Pain	Age, numeric rating scale, and biological effective dose 10	*RF*	LOOCV
Zhou et al [36]	China, 2021	Clinical data; 386	People with colorectal cancer after chemotherapy	Cognitive impairment	Age, BMI, colostomy, treatment complications, cancer-related anemia, depression, diabetes, Quality of Life Questionnaire Core 30 score, exercise, hypercholesterolemia, diet, marital status, education level, and pathological stage	RF, *LR,* and SVM	Random
Xuyi et al [37]	Canada, 2021	Clinical data; 46,104	Specific cancer site or treatment not mentioned	Pain, depression, and well-being	Lung cancer, late-stage cancer, existing chronic conditions such as osteoarthritis, mood disorder, hypertension, diabetes, and coronary disease	*ANN*	Random
Xu et al [38]	China, 2021	Clinical data; 598	People with gastrointestinal tumors after surgery	Postoperative fatigue	Age, higher degree of education, lower personal monthly income, advanced cancer, hypoproteinemia, preoperative anxiety or depression, and limited social support	LR, *ANN*, CART^w^	Random
Wei et al [39]	China, 2021	Clinical data; 533	People with breast cancer	Lymphedema	N/A	ANN, *LR*, C5.0, RF, SVM, CART	10-fold CV
Wang et al [40]	United States, 2021	Clinical data; 823	People with HNC	Pain, taste, and general activity	N/A	SVM, KNN, and RF; Gaussian NB and MLP^x^; and ARIMA^y^ and *LSTM^z^*	Random
Wang et al [41]	United States, 2021	Clinical data and CT image data; 138	Specific cancer site or treatment not mentioned	Depression	N/A	*Fine tree*, *medium tree*, coarse tree, linear-discriminant, quadratic discriminant, LR, Gaussian NB, kernel NB, linear SVM, quadratic SVM, cubic SVM, Fine Gaussian SVM, Medium Gaussian SVM, Coarse Gaussian SVM, Fine KNN, Medium KNN, Coarse KNN, Cosine KNN, Cubic KNN, Weighted KNN, boosted trees, bagged trees, subspace discriminant, subspace KNN, and random undersampling boosted trees	5-fold CV
Mosa et al [17]	United States, 2021	Clinical data; 6124	People with cancer receiving chemotherapy	Nausea-vomiting	Smoking, alcohol status, sex, age, and BMI	NB, LR, ANN, SVR^aa^, and *DT*	10-fold CV
Low et al [42]	United States, 2021	Clinical data; 44	People with pancreatic cancer after surgery	Diarrhea, fatigue, and pain	Physical activity bouts, sleep, heart rate, and location	LR, KNN, SVM, RF, GB^ab^, XGB, and *LightGBM*	3-fold CV and LOOCV
Kourou et al [43]	Greece, 2021	Clinical data; 609	People with breast cancer	Depression	A set of psychological traits (optimism, perceived ability to cope with trauma, resilience as a trait, and ability to understand the illness) and subjective perceptions of personal functionality (physical, social, and cognitive)	RF, *SVM*, and GB	5-fold CV
Kober et al [44]	United States, 2021	Clinical data; 1217	People with cancer receiving chemotherapy	Evening fatigue	Morning fatigue, lower evening energy, and sleep disturbance	*RF*, LR (filtered or unfiltered), RPAR, and SVM	10-fold CV
Hu et al [45]	China, 2021	Clinical data; 238	People with non-Hodgkin lymphoma receiving chemotherapy	Depression	Education level, sex, age, marital status, medical insurance, per capita monthly household income, pathological stage, Suicide Severity Rating Scale, Pittsburgh Sleep Quality Index, and Quality of Life Questionnaire Core 30	SVM, RF, and *LASSO+LR*	Random
Haun et al [46]	Germany, 2021	Clinical data; 496	People with cancer seen in primary care	Anxiety	Fatigue or weakness, insomnia, and pain appeared	OLS^ac^, RR^ad^, *LASSO*, ENR^ae^, RF, and XGB	10-fold CV
Lee et al [47]	United States, 2020	Clinical data and CT Images data; 388	People with lung cancer after intensity-modulated radiation therapy	Weight loss	Joint Gross tumor volume L1+L2+L3 radiomics, Gross tumor volume, and esophagus L3 dosiomic	SVM, DNN^af^, and *ensemble classifier*	Nested CV
Juwara et al [48]	Canada, 2020	Clinical data; 204	People with breast cancer after surgery	NP^aj^	Anxiety, type of surgery, and acute pain	LS^ah^, *RR*, *ENR*, RF, *GB*, and ANN	10-fold CV
Men et al [49]	United States, 2019	Clinical data and CT image data; 784	People with HNC receiving radiotherapy	Xerostomia	Feature map visualization	LR and *3D-RCNN^ai^*	Random
Jiang et al [50]	United States, 2019	Clinical data and CT images data; 427	People with HNC	Xerostomia	The patient has human papillomavirus, completed chemotherapy, their baseline xerostomia grade, tumor site, N stage, and use of feeding tube	*RR*, LASSO, and RF	10-fold CV
Sheikh et al [51]	United States, 2019	CT images data; 266	People with HNC	Xerostomia	N/A	*Generalized linear model*	10-fold CV
Papachristou et al [52]	United States, 2019	Clinical data; 799	People with cancer receiving chemotherapy	Sleep disturbance, anxiety, and depression	Age, gender, cancer site, the number of prior cancer treatment, and initial diagnosis	*SVR* (linear, polynomial, and radial Sigma) and n-CCA^aj^	10-fold CV and bootstrap
Zhang et al [53]	China, 2018	Clinical data; 375	People with cancer receiving radiotherapy	Weight loss	Head and neck tumor location and total radiation dose of ≥70 Gray, and without postsurgery	*DT* and LR	Random
Olling et al [54]	Denmark;2018	Clinical and CT image; 131	People with lung cancer receiving radiotherapy	Odynophagia (painful swallowing)	N/A	*Multivariable LR***,** Lasso and elastic net regularized generalized linear models, and *SVM*	10-fold CV
Gabryś et al [55]	Germany;2018	Clinical and CT image; 153	People with HNC after radiotherapy	Xerostomia	The parotid gland volume, the spread of the contralateral dose-volume histogram, and the parotid gland eccentricity, and sex	LRL1^ak,^ LRL2^al^, LR-EN^am^, *KNN,* SVM, *ET^an^*, and *GTB^ao^*	Single and nested CV
Lötsch et al [56]	Germany;2018	Clinical data; 1000	People with breast cancer after surgery	Pain	Age, chronic pain of any type, number of previous operations, BMI, preoperative pain in the area to be operated on, smoking and psychological factors	*Unsupervised ML^ap^*	Random
Abdollahi et al [57]	Iran;2018	Clinical and CT image; 47	People with HNC receiving chemotherapy	Hearing loss	10 of the 490 radiomic features selected as the associated features with significant sensorineural hearing loss status	Decision stump, Hoeffding, C4.5, NB, AdaBoost, bootstrap aggregating, and LR	10-fold CV
van Dijk et al [58]	United States;2018	Clinical data and CT image; 68	People with HNC	Xerostomia	N/A	*LR*	External validation
Cvetković [59]	Serbia;2017	Clinical data; 84	People with breast cancer	Depression	N/A	*ELM^aq^*, ANN, and Fuzzy Genetic Algorithm	Random
van Dijk et al [60]	United States;2017	CT image features; 249	People with HNC	Xerostomia	N/A	*LR*	10-fold CV

^a^LR: logistic regression.

^b^Italic text in this column indicates the best results used in the study.

^c^RF: random forest.

^d^GBDT: gradient boosting decision tree.

^e^XGB: extreme gradient boosting.

^f^ANN: artificial neural network.

^g^CV: cross-validation.

^h^EN: elastic net.

^i^LASSO: Least absolute shrinkage and selection operator.

^j^RPAR: recursive partitioning and regression trees.

^k^SVM: support vector machine.

^l^NB: Naïve bayes.

^m^N/A: not applicable.

^n^MP: multiple perceptron.

^o^AdaBoost: Adaptive boosting.

^p^GBM: light gradient boosting machine.

^q^CT: computed tomography.

^r^CNN: convolutional neural network.

^s^DT: decision tree.

^t^LOOCV: leave-one-out-cross-validation.

^u^HNC: head and neck cancer.

^v^KNN: k-nearest neighbor.

^w^CART: classification and regression tree.

^x^MLP: multilayer perceptron.

^y^ARIMA: autoregressive integrated moving average.

^z^LSTM: long short-term memory neural network.

^aa^SVR: support vector regression.

^ab^GB: gradient boosting.

^ac^OLS: ordinary least square.

^ad^RR: ridge regression.

^ae^ENR: elastic net regression.

^af^DNN: deep neural network.

^ag^NP: neuropathic pain.

^ah^LS: least squares.

^ai^3D-RCNN: 3D region-based convolutional neural network.

^aj^n-CCA: nonlinear canonical correlation analysis.

^ak^LRL1: L1 penalized logistic regression.

^al^LRL2: L2 penalized logistic regression.

^am^LR-EN: logistic regression-elastic net.

^an^ET: extra tree.

^ao^GTB: gradient tree boosting.

^ap^ML: machine learning.

^aq^ELM: extreme linear machine.

A total of 2 individual researchers (NZ and NY) separately extracted data from each study, working independently of each other. This approach is used to reduce bias and increase the accuracy of the data extraction process. If discrepancies arise between the 2 independent authors, they are usually resolved through discussion or by consulting a third reviewer (SGW).

### Primary Database Information

The studies selected were published between 2017 and 2023 and were conducted in North America (18/42, 43%), Asia (16/42, 38%), and Europe (8/42, 19%). Methods of data collection varied, with studies originating from individual centers (23/42, 55%) and multiple centers (19/42, 45%). The average sample size was 1686, and the studies varied in sample size: <100 participants (8/42, 19%), between 100 and 1000 participants (27/42, 64%), and >1000 participants (7/42, 17%). Most studies relied on clinical data (28/42, 67%), although some integrated clinical data with computed tomography (CT) images (14/42, 33%). The study designs were diverse, including retrospective (18/42, 43%), cross-sectional (15/42, 38%), prospective (5/42, 12%), and longitudinal (4/42, 10%) approaches.

### Cancer Primary Sites and Predicted Symptoms

Various primary cancer sites were studied, with head and neck cancers being the most prevalent (9/42, 21%). Breast cancer was the focus of 19% (8/42) of the studies, and lung cancer was studied in 17% (3/42) of the cases. The included studies included participants undergoing a range of treatments, including chemotherapy (9/42, 21%), radiotherapy (9/42, 21%), surgery (4/42, 10%), and investigations of posttreatment survivors (2/42, 5%). Of the 42 included studies, 10 unique symptoms were reported as outcome variables in the predictions. Those included were xerostomia (9/42, 14%) [27,30,34,49-51,55,58,60], depression (8/42, 13%) [22,33,37,41,43,45,52,59], pain (8/42, 13%) [20,25,26,35,37,40,42,56], fatigue (6/42, 10%) [23,24,29,38,42,44], anxiety (3/42, 5%) [33,46,52], sleep disturbance or insomnia (3/42, 5%) [26,28,52], nausea or vomiting (3/42, 5%) [17,26,29], weight loss (2/42, 3%) [47,53], cognitive impairment (2/42, 3%) [21,36], and diarrhea (2/42, 3%) [29,42].

One study reported multiple symptoms, including hypersensitivity [29], stomatitis [29], hand-foot syndrome [29], peripheral neuropathy [29], and constipation [29]. Another study delved into taste and general activity [40]. Individual studies were dedicated to each of the following symptoms: delirium [31], lung infection [32], lymphedema [39], well-being [37], odynophagia [54], social distress [26], spiritual pain [26], dyspnea [26], and hearing loss [57]. The distribution of these symptoms is depicted in Multimedia Appendix 5.

### Significant Candidate Predictors of Symptoms

Numerous predictors were frequently used for predicting symptoms, which can be grouped into demographic features and clinical characteristics.

#### Demographic Features

The demographic features include age, sex, BMI, income, medical insurance, education, marital status, and zip code–level poverty.

#### Clinical Characteristics

The clinical characteristics include smoking and alcohol use, initial diagnosis, presence of cancer, stage of cancer, cancer course, tumor site, type and number of prior treatments, chemotherapy type, and radiotherapy dose and volume. Health conditions such as comorbidity, diabetes, hypertension, osteoarthritis, and coronary disease also play a significant role. In addition, psychological factors such as depression and anxiety, fatigue, sleep disturbance, and pain are considered. Other influential predictors encompass care fragmentation, polypharmacy, hormone levels, physical activity, diet, heart rate, and social support factors.

**Figure 2:**
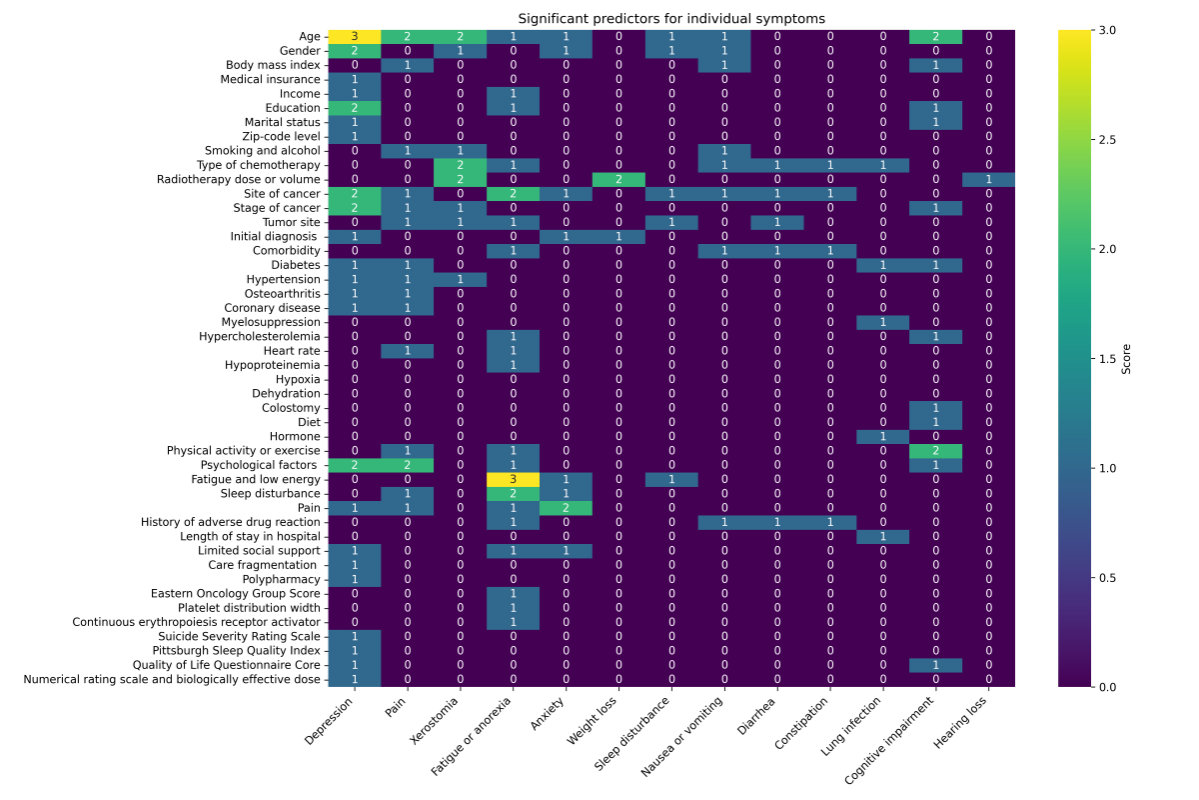
Significant predictors of individual symptoms.

In our comprehensive analysis of 42 studies, all the detailed findings on common cancer symptoms are compiled in Figure 2. We provide a detailed analysis of the predictors for the 4 most frequently reported cancer symptoms identified in this study: xerostomia, pain, depression, and fatigue. In a detailed analysis of 42 studies, various predictors for 4 common cancer symptoms—xerostomia, pain, depression, and fatigue—have been identified, each with its distinct set of influencing factors.

For xerostomia, age, gender, chemotherapy type, radiotherapy dose and volume, cancer stage, tumor site, and hypertension are crucial predictors. In the case of pain, factors such as age, BMI, smoking and alcohol habits, cancer site and stage, tumor site, diabetes, hypertension, osteoarthritis, coronary disease, physical activity, psychological factors, sleep disorders, and existing pain conditions emerge as significant. Significant predictors for depression include age; gender; education; cancer site and stage; economic factors such as insurance, income, and poverty level; marital status; initial diagnosis impact; comorbidities (diabetes, hypertension, osteoarthritis, and coronary disease); pain; social support; care fragmentation; polypharmacy; and various scale scores. Finally, for fatigue, the key predictors are existing fatigue and low energy, cancer site, sleep disturbances, age, income, education, chemotherapy type, tumor site, comorbidities, hypercholesterolemia, heart rate, hypoproteinemia, physical and psychological factors, pain, adverse drug reaction history, limited social support, Eastern Cooperative Oncology Group score, platelet distribution width, and erythropoiesis.

When examining the commonalities across these predictors for xerostomia, pain, depression, and fatigue, several factors stand out as particularly influential across multiple symptoms: age; gender; cancer site and stage; treatment-related factors such as the type of chemotherapy and radiotherapy; comorbidities such as diabetes, hypertension, and coronary disease; physical and psychological factors; and socioeconomic factors such as income and education level, demonstrating the impact of cancer treatments on symptom development. These common predictors underscore the complex, multifactorial nature of symptom manifestation in patients with cancer, necessitating a comprehensive approach to their management and care.

### ML Algorithms and Validation Metrics

Of the 42 studies analyzed, 7 (17%) used a single ML algorithm, whereas 35 (83%) used multiple algorithms. The most effective models, in terms of performance, were logistic regression (LR; 9/42, 17%), random forest (7/42, 13%), artificial neural networks (5/42, 9%), decision trees (DTs; 5/42, 9%), and extreme gradient boosting (3/42, 6%). For validation methods, 10-fold cross-validation was the most used (14/42, 31%), followed by 5-fold cross-validation (5/42, 11%), 3-fold cross-validation (2/42, 4%), and 8-fold cross-validation (1/42, 2%). The primary evaluation metric across these studies was the area under the curve, which was adopted in 24% (26/42) of the studies. A visual representation of the leading ML models along with the validation and evaluation metrics used in the study presents in Multimedia Appendix 6.

## Discussion

### Principal Findings

In this review, we present the first systematic analysis of ML applications for predicting the development of cancer symptoms. We explore the most frequently studied cancer sites and delve into the intricacies of ML procedures. Breast, head or neck, and lung cancers are the most frequently studied sites in current research, with xerostomia, depression, pain, and fatigue being the most prominent symptoms. The application of various ML techniques is on the rise, with data acquisition and preprocessing being pivotal for successful ML models. While a range of algorithms, from traditional methods such as LR and DT to advanced ones such as DL, are used, there is a growing emphasis on data quality, external validation, and a standardized approach to model evaluation. The future of ML in cancer symptom prediction looks promising, with a need for collaborative efforts among oncologists, data scientists, and patient groups, combined with more comprehensive research on lesser-studied cancer sites and standardized methodologies.

Regarding the cancer sites covered in the studies, breast, head or neck, and lung cancers emerged as the most frequently researched primary cancer sites. The range of symptoms and side effects that patients experienced varied from one study to another. Some symptoms depended on the specific cancer site and the treatments patients received. For example, xerostomia, which can either arise from the tumor itself or manifest as a treatment side effect, has a significant impact on patients’ dental health and compromises antimicrobial functions [61]. However, most symptoms were not directly attributed to a particular cancer site or treatment.

Our review revealed a notable emphasis on predicting xerostomia in 14% (9/42) of the studies, despite head and neck cancers being less prevalent. The notable emphasis on predicting xerostomia in ML research, despite the lower prevalence of head and neck cancers, is likely due to advancements in integrating ML with CT imaging. CT imaging is a pivotal tool in the diagnosis and treatment planning of head and neck cancers. The integration of ML with CT imaging has opened new possibilities for more accurately predicting side effects such as xerostomia. ML techniques, when applied to CT images, can potentially identify patterns and indicators that are not easily discernible by human observers. This capability can lead to earlier and more precise predictions of xerostomia, thereby enabling better preventive measures and treatment planning to mitigate this side effect. Therefore, the focus on xerostomia in ML research, in the context of head and neck cancers, is likely driven by the opportunities presented by combining ML with advanced imaging techniques.

Depression, a widespread emotional challenge for people with cancer [62,63], was the focus of prediction in many studies (8/24, 13%). Similarly, pain, a recurrent concern for palliative care patients [64] and survivors of cancer [65,66], was the subject of prediction in >13% (8/24) of the studies. Fatigue, prevalent across all age groups with cancer [67,68], was highlighted in 6 (10%) of the 42 studies reviewed.

In terms of the ML approaches used in the studies, a plethora of techniques were used to construct these predictive models, spanning all phases of the ML process, from data collection and preprocessing to feature and algorithm selection, model training, testing, and evaluation. The process of data acquisition is pivotal for the development of ML models, thereby emphasizing the importance of an adequate sample size. Upon reviewing 42 studies, we discerned that the most frequent sample sizes for ML applications ranged between 100 and 1000 samples. More advanced ML techniques necessitate larger data sets to bolster robustness and mitigate the risk of overfitting. Alarmingly, certain studies in our review used ML with comparably smaller data sets, introducing the risk of model overfitting and potential biases in the subsequent performance metrics [69]. Challenges tied to sample size might impede the creation of sturdy and trustworthy ML models [70]. Data preprocessing is indispensable to yield clean and interpretable data, which is a cornerstone for proficient ML models. Data cleaning approaches encompass addressing missing values, tackling data noise, and data normalization. Within health care data sets, noisy or absent data are frequently a by-product of inaccuracies in manual entries or instrument recordings made by medical personnel or ancillary staff [71]. However, most of the reviewed studies lacked comprehensive descriptions of their data cleaning methodologies or strategies for handling noisy data and normalization, constrained by word or page limits in publications.

Given the crucial importance of data quality in developing ML models, it is essential for researchers to focus equally on effective data preparation and choosing suitable algorithms. Future endeavors would benefit from exhaustive procedural documentation made available on public platforms such as GitHub. In a research context, GitHub can be used for sharing and collaborating on various aspects of a research project, including but not limited to code. It allows researchers to maintain version control of their scripts, data analysis procedures, and even documentation. This feature is particularly beneficial for replicating studies and verifying results, as it provides a transparent view of the methodologies and analyses used.

Overloading an ML model with excessive features can undermine its ability to differentiate between pertinent data and superfluous noise, leading to the challenge often referred to as the “curse of dimensionality.” The goal of feature engineering is to mitigate model complexity, expedite the training process, reduce the data’s dimensionality, and avert overfitting [72]. By streamlining the model with a curated set of predictors, it becomes more accessible and transparent, emphasizing the importance of feature selection during data preparation. Our review pinpointed the most frequently used significant predictors in cancer symptom prediction. The efficacy of prediction models is heavily influenced by the number and interplay of the relevant predictors. Factors such as age, gender, type and number of previous treatments, cancer location, cancer stage, chemotherapy type, dosage and volume of radiotherapy; chronic conditions such as diabetes and hypertension; concurrent diseases; and symptoms including depression, anxiety, fatigue, pain, and sleep disturbances have consistently featured as determinants in numerous predictive frameworks. Our review of cancer symptom prediction underscored age as a pivotal factor, associated with predominant symptoms such as depression, pain, xerostomia, and fatigue. While numerous elements, from gender to type of treatment and cancer stage, influence the predictive models, it is the prominence of age that consistently emerges as a cornerstone predictor. As we delve deeper into this field, even with the introduction of newer determinants and correlations, the centrality of age in these frameworks remains indisputable.

Regarding algorithm selection, traditional methods often struggle with handling high-dimensional data and processing extensive information. To tackle these challenges, researchers have increasingly shifted toward innovative ML algorithms that are renowned for their robust predictive power and strong generalization capacities. These sophisticated algorithms excel at delving deep into data and discerning intricate interrelationships among variables. To navigate the multifaceted landscape of modeling challenges, it is advantageous for researchers to leverage a diverse array of ML algorithms. Most studies used multiple predictive models, with techniques such as LR, RF, ANN, and DT consistently delivering stellar results. The introduction of advanced ML techniques, such as DL and ensemble classifiers, provides promising opportunities to elevate prediction accuracy in future research.

After their design, the ML models undergo training and testing on different data sets. However, these models can grapple with issues such as overfitting and underfitting. Overfitting occurs when a model becomes overly complex, which leads to increased variance and reduced clarity. In contrast, underfitting results from an oversimplified model, causing it to overlook key data patterns and diminish its predictive capacity. Therefore, the ideal learning model should strike a balance between the optimal variance and justifiable bias. To mitigate these issues, the common strategy is to divide the data set into training and testing subsets, followed by internal or external validation. While most studies in our review used internal validation, only 1 study reported external validation [58], which was demonstrated on a small cohort of 25 patients with head and neck cancer. Although its performance is typically lower than evaluations using the original data sets, external validation remains crucial for gauging ML models [72]. It is a crucial step in ensuring that the model’s performance is not just limited to the conditions and data it was originally trained on but also applicable and reliable in broader, real-world clinical settings. This approach serves to verify the model’s efficacy and generalizability across different patient populations and settings.

Understanding and interpreting ML models continue to pose challenges. Determining the variables that significantly impact symptom prediction can be elusive due to the intricate prediction processes. Many studies gauge the performance of ML models using metrics that examine their ability to distinguish between 2 classes. From our systematic review of 42 studies, the area under the curve emerged as the predominant metric for the prediction models. Other metrics included accuracy, sensitivity, specificity, positive predictive value, root mean square error, and negative predictive value. These metrics provide a holistic view of a model’s efficacy, facilitating its refinement and enabling more precise predictions. However, the diverse emphasis on distinct metrics in numerous studies underscores the need for a uniform approach to evaluating ML models in cancer symptom prediction.

As interest grows in using ML for predicting cancer symptoms, there are several areas that merit deeper investigation. A crucial area is broadening the range of studied cancer sites and more comprehensively correlating symptoms with various treatment methods. To fully understand symptom prediction, it is essential that future studies delve into lesser-explored or infrequently studied cancer sites. Furthermore, the methodologies used for data preprocessing and cleaning should be documented more thoroughly, focusing on best practices to ensure data integrity. As data are foundational to ML models, transparent and detailed preprocessing can improve the reliability and repeatability of these models. Although our analysis highlighted common predictors for symptom forecasting, examining potentially underrepresented or emerging indicators could refine these models further. On the algorithmic front, exploring hybrid ML methods that merge the strengths of multiple algorithms might be particularly beneficial for cancer symptom prediction. Standardizing evaluation metrics across studies would also provide clarity and facilitate a more accurate comparison of various ML techniques. To genuinely progress, collaborations among oncologists, data scientists, and patient advocacy groups are vital to ensure that the developed models are technically robust and clinically pertinent. With these insights, ML stands poised to transform cancer care, creating treatment plans based on patient-focused and accurate symptom prediction models.

### Limitations

This review is not without its limitations. Although we established clear inclusion and exclusion criteria, potential biases in the studies we analyzed could inherently limit our review. We might have missed or excluded relevant studies due to inadequate information or the absence of keywords in their titles or abstracts. Many of the studies we reviewed did not specify the cancer site, potentially limiting the accuracy and applicability of our findings to specific cancer types. The broad range of predictors used across the studies also made it difficult to draw definitive conclusions about the most influential factors in predicting cancer symptoms using ML algorithms. As such, readers should interpret these results cautiously, given this variability.

### Conclusions

ML offers an intriguing potential for predicting cancer symptoms, thereby preemptively mitigating the associated challenges. Predicting the symptoms that people with cancer might experience and determining their onset throughout their treatment journey is a pivotal clinical issue that can enhance patients’ quality of life. Notably, all studies in our review were published after 2017, highlighting the nascent nature of this research area. Our investigation primarily sought to outline the ML methodologies harnessed for symptom prediction in people with cancer. While ML techniques hold an edge over traditional statistical approaches by virtue of their prowess in analyzing vast data sets and gauging the efficacy of diverse prediction models, certain impediments such as a limited pool of symptoms; suboptimal data preparation; challenges in feature engineering; and complexities in ML algorithm design, validation, and evaluation can constrain the broad applicability of these predictive models. Future research should pivot toward amplifying the efficacy of ML strategies. This enhancement can be achieved by harnessing expansive, high-caliber data sets; tapping into innovative technologies for data refinement; and sculpting refined models. Harnessing ML can potentially free health care practitioners—including doctors, nurses, and clinic personnel—to accentuate the human touch in managing cancer symptoms.

## Appendix

Multimedia Appendix 1 The detailed search strategy for the databases and the Boolean expressions used.

Multimedia Appendix 2 PRISMA (Preferred Reporting Items for Systematic Reviews and Meta-Analyses) 2020 checklist.

Multimedia Appendix 3 The distribution and overlap of 42 studies across the databases.

Multimedia Appendix 4 The data extracted from 42 studies.

Multimedia Appendix 5 Number of studies per cancer symptoms.

Multimedia Appendix 6 Visual overview of the machine learning models and metrics.

## Abbreviations

CT: computed tomography
DL: deep learning
DT: decision tree
LR: logistic regression
ML: machine learning
PRISMA: Preferred Reporting Items for Systematic Reviews and Meta-Analyses

## References

[1] Sung H, Ferlay J, Siegel RL, Laversanne M, Soerjomataram I, Jemal A, Bray F (2021). Global cancer statistics 2020: GLOBOCAN estimates of incidence and mortality worldwide for 36 cancers in 185 countries. CA Cancer J Clin.

[2] Yates P (2017). Symptom management and palliative care for patients with cancer. Nurs Clin North Am.

[3] Abd El-Aziz N, Khallaf S, Abozaid W, Elgohary G, Abd El-Fattah O, Alhawari M, Khaled S, AbdelHaffez A, Kamel E, Mohamed S (2020). Is it the time to implement the routine use of distress thermometer among Egyptian patients with newly diagnosed cancer?. BMC Cancer.

[4] Abu-Odah H, Molassiotis A, Yat Wa Liu J (2022). Analysis of the unmet needs of Palestinian advanced cancer patients and their relationship to emotional distress: results from a cross-sectional study. BMC Palliat Care.

[5] Martínez Arroyo O, Andreu Vaíllo Y, Martínez López P, Galdón Garrido MJ (2019). Emotional distress and unmet supportive care needs in survivors of breast cancer beyond the end of primary treatment. Support Care Cancer.

[6] Saeidzadeh S, Kamalumpundi V, Chi N, Nair R, Gilbertson-White S (2021). Web and mobile-based symptom management interventions for physical symptoms of people with advanced cancer: A systematic review and meta-analysis. Palliat Med.

[7] Cleeland CS, Bennett GJ, Dantzer R, Dougherty PM, Dunn AJ, Meyers CA, Miller AH, Payne R, Reuben JM, Wang XS, Lee B (2003). Are the symptoms of cancer and cancer treatment due to a shared biologic mechanism? A cytokine-immunologic model of cancer symptoms. Cancer.

[8] Cleeland CS (2007). Symptom burden: multiple symptoms and their impact as patient-reported outcomes. J Natl Cancer Inst Monogr.

[9] Kaasa S, Loge JH, Aapro M, Albreht T, Anderson R, Bruera E, Brunelli C, Caraceni A, Cervantes A, Currow DC, Deliens L, Fallon M, Gómez-Batiste X, Grotmol KS, Hannon B, Haugen DF, Higginson IJ, Hjermstad MJ, Hui D, Jordan K, Kurita GP, Larkin PJ, Miccinesi G, Nauck F, Pribakovic R, Rodin G, Sjøgren P, Stone P, Zimmermann C, Lundeby T (2018). Integration of oncology and palliative care: a Lancet oncology commission. Lancet Oncol.

[10] Hunter B, Hindocha S, Lee RW (2022). The role of artificial intelligence in early cancer diagnosis. Cancers (Basel).

[11] Ruffle JK, Farmer AD, Aziz Q (2019). Artificial intelligence-assisted gastroenterology- promises and pitfalls. Am J Gastroenterol.

[12] Ahmed H, Soliman H, Elmogy M (2022). Early detection of Alzheimer's disease using single nucleotide polymorphisms analysis based on gradient boosting tree. Comput Biol Med.

[13] Abouzari M, Goshtasbi K, Sarna B, Khosravi P, Reutershan T, Mostaghni N, Lin HW, Djalilian HR (2020). Prediction of vestibular schwannoma recurrence using artificial neural network. Laryngoscope Investig Otolaryngol.

[14] Liu YH, Jin J, Liu YJ (2022). Machine learning-based random forest for predicting decreased quality of life in thyroid cancer patients after thyroidectomy. Support Care Cancer.

[15] van de Wiel M, Derijcke S, Galdermans D, Daenen M, Surmont V, De Droogh E, Lefebure A, Saenen E, Vandenbroucke E, Morel A, Sadowska A, van Meerbeeck JP, Janssens A (2021). Coping strategy influences quality of life in patients with advanced lung cancer by mediating mood. Clin Lung Cancer.

[16] Aafjes-van Doorn K, Kamsteeg C, Bate J, Aafjes M (2021). A scoping review of machine learning in psychotherapy research. Psychother Res.

[17] Mosa AS, Rana MK, Islam H, Hossain AK, Yoo I (2021). A smartphone-based decision support tool for predicting patients at risk of chemotherapy-induced nausea and vomiting: retrospective study on app development using decision tree induction. JMIR Mhealth Uhealth.

[18] Geron A (2019). Hands-On Machine Learning with Scikit-Learn, Keras, and TensorFlow: Concepts, Tools, and Techniques to Build Intelligent Systems.

[19] Tam WW, Tang A, Woo B, Goh SY (2019). Perception of the preferred reporting items for systematic reviews and meta-analyses (PRISMA) statement of authors publishing reviews in nursing journals: a cross-sectional online survey. BMJ Open.

[20] Sun C, Li M, Lan L, Pei L, Zhang Y, Tan G, Zhang Z, Huang Y (2023). Prediction models for chronic postsurgical pain in patients with breast cancer based on machine learning approaches. Front Oncol.

[21] Xinran Z, Shumei Z, Xueying Z, Linan W, Ying G, Peng W, Yahong H, Longting M, Jing W (2023). Construction of a predictive model for cognitive impairment risk in patients with advanced cancer. Int J Nurs Pract.

[22] Shaikh NF, Shen C, LeMasters T, Dwibedi N, Ladani A, Sambamoorthi U (2023). Prescription non-steroidal anti-inflammatory drugs (NSAIDs) and incidence of depression among older cancer survivors with osteoarthritis: a machine learning analysis. Cancer Inform.

[23] Kober KM, Roy R, Conley Y, Dhruva A, Hammer MJ, Levine J, Olshen A, Miaskowski C (2023). Prediction of morning fatigue severity in outpatients receiving chemotherapy: less may still be more. Support Care Cancer.

[24] Du L, Du J, Yang M, Xu Q, Huang J, Tan W, Xu T, Wang L, Nie W, Zhao L (2023). Development and external validation of a machine learning-based prediction model for the cancer-related fatigue diagnostic screening in adult cancer patients: a cross-sectional study in China. Support Care Cancer.

[25] Moscato S, Orlandi S, Giannelli A, Ostan R, Chiari L (2022). Automatic pain assessment on cancer patients using physiological signals recorded in real-world contexts. Annu Int Conf IEEE Eng Med Biol Soc.

[26] Masukawa K, Aoyama M, Yokota S, Nakamura J, Ishida R, Nakayama M, Miyashita M (2022). Machine learning models to detect social distress, spiritual pain, and severe physical psychological symptoms in terminally ill patients with cancer from unstructured text data in electronic medical records. Palliat Med.

[27] Fanizzi A, Scognamillo G, Nestola A, Bambace S, Bove S, Comes MC, Cristofaro C, Didonna V, Di Rito A, Errico A, Palermo L, Tamborra P, Troiano M, Parisi S, Villani R, Zito A, Lioce M, Massafra R (2022). Transfer learning approach based on computed tomography images for predicting late xerostomia after radiotherapy in patients with oropharyngeal cancer. Front Med (Lausanne).

[28] Ueno T, Ichikawa D, Shimizu Y, Narisawa T, Tsuji K, Ochi E, Sakurai N, Iwata H, Matsuoka YJ (2022). Comorbid insomnia among breast cancer survivors and its prediction using machine learning: a nationwide study in Japan. Jpn J Clin Oncol.

[29] On J, Park HA, Yoo S (2022). Development of a prediction models for chemotherapy-induced adverse drug reactions: a retrospective observational study using electronic health records. Eur J Oncol Nurs.

[30] Li M, Zhang J, Zha Y, Li Y, Hu B, Zheng S, Zhou J (2022). A prediction model for xerostomia in locoregionally advanced nasopharyngeal carcinoma patients receiving radical radiotherapy. BMC Oral Health.

[31] Kurisu K, Inada S, Maeda I, Ogawa A, Iwase S, Akechi T, Morita T, Oyamada S, Yamaguchi T, Imai K, Nakahara R, Kaneishi K, Nakajima N, Sumitani M, Yoshiuchi K, Phase-R Delirium Study Group (2022). A decision tree prediction model for a short-term outcome of delirium in patients with advanced cancer receiving pharmacological interventions: a secondary analysis of a multicenter and prospective observational study (Phase-R). Palliat Support Care.

[32] Guo W, Gao G, Dai J, Sun Q (2022). Prediction of lung infection during palliative chemotherapy of lung cancer based on artificial neural network. Comput Math Methods Med.

[33] Baglione AN, Cai L, Bahrini A, Posey I, Boukhechba M, Chow PI (2022). Understanding the relationship between mood symptoms and mobile app engagement among patients with breast cancer using machine learning: case study. JMIR Med Inform.

[34] Chao M, El Naqa I, Bakst RL, Lo Y, Peñagarícano JA (2022). Cluster model incorporating heterogeneous dose distribution of partial parotid irradiation for radiotherapy induced xerostomia prediction with machine learning methods. Acta Oncol.

[35] Wakabayashi K, Koide Y, Aoyama T, Shimizu H, Miyauchi R, Tanaka H, Tachibana H, Nakamura K, Kodaira T (2021). A predictive model for pain response following radiotherapy for treatment of spinal metastases. Sci Rep.

[36] Zhou SP, Fei SD, Han HH, Li JJ, Yang S, Zhao CY (2021). A prediction model for cognitive impairment risk in colorectal cancer after chemotherapy treatment. Biomed Res Int.

[37] Xuyi W, Seow H, Sutradhar R (2021). Artificial neural networks for simultaneously predicting the risk of multiple co-occurring symptoms among patients with cancer. Cancer Med.

[38] Xu XY, Lu JL, Xu Q, Hua HX, Xu L, Chen L (2021). Risk factors and the utility of three different kinds of prediction models for postoperative fatigue after gastrointestinal tumor surgery. Support Care Cancer.

[39] Wei X, Lu Q, Jin S, Li F, Zhao Q, Cui Y, Jin S, Cao Y, Fu MR (2021). Developing and validating a prediction model for lymphedema detection in breast cancer survivors. Eur J Oncol Nurs.

[40] Wang Y, Van Dijk L, Mohamed AS, Fuller CD, Zhang X, Marai GE, Canahuate G (2021). Predicting late symptoms of head and neck cancer treatment using LSTM and patient reported outcomes. Proc Int Database Eng Appl Symp.

[41] Wang X, Eichhorn J, Haq I, Baghal A (2021). Resting-state brain metabolic fingerprinting clusters (biomarkers) and predictive models for major depression in multiple myeloma patients. PLoS One.

[42] Low CA, Li M, Vega J, Durica KC, Ferreira D, Tam V, Hogg M, Zeh Iii H, Doryab A, Dey AK (2021). Digital biomarkers of symptom burden self-reported by perioperative patients undergoing pancreatic surgery: prospective longitudinal study. JMIR Cancer.

[43] Kourou K, Manikis G, Poikonen-Saksela P, Mazzocco K, Pat-Horenczyk R, Sousa B, Oliveira-Maia AJ, Mattson J, Roziner I, Pettini G, Kondylakis H, Marias K, Karademas E, Simos P, Fotiadis DI (2021). A machine learning-based pipeline for modeling medical, socio-demographic, lifestyle and self-reported psychological traits as predictors of mental health outcomes after breast cancer diagnosis: an initial effort to define resilience effects. Comput Biol Med.

[44] Kober KM, Roy R, Dhruva A, Conley YP, Chan RJ, Cooper B, Olshen A, Miaskowski C (2021). Prediction of evening fatigue severity in outpatients receiving chemotherapy: less may be more. Fatigue.

[45] Hu C, Li Q, Shou J, Zhang F, Li X, Wu M, Xu M, Xu L (2021). Constructing a predictive model of depression in chemotherapy patients with Non-Hodgkin's lymphoma to improve medical staffs' psychiatric care. Biomed Res Int.

[46] Haun MW, Simon L, Sklenarova H, Zimmermann-Schlegel V, Friederich H, Hartmann M (2021). Predicting anxiety in cancer survivors presenting to primary care - a machine learning approach accounting for physical comorbidity. Cancer Med.

[47] Lee SH, Han P, Hales RK, Voong KR, Noro K, Sugiyama S, Haller JW, McNutt TR, Lee J (2020). Multi-view radiomics and dosiomics analysis with machine learning for predicting acute-phase weight loss in lung cancer patients treated with radiotherapy. Phys Med Biol.

[48] Juwara L, Arora N, Gornitsky M, Saha-Chaudhuri P, Velly AM (2020). Identifying predictive factors for neuropathic pain after breast cancer surgery using machine learning. Int J Med Inform.

[49] Men K, Geng H, Zhong H, Fan Y, Lin A, Xiao Y (2019). A deep learning model for predicting xerostomia due to radiation therapy for head and neck squamous cell carcinoma in the RTOG 0522 clinical trial. Int J Radiat Oncol Biol Phys.

[50] Jiang W, Lakshminarayanan P, Hui X, Han P, Cheng Z, Bowers M, Shpitser I, Siddiqui S, Taylor RH, Quon H, McNutt T (2019). Machine learning methods uncover radiomorphologic dose patterns in salivary glands that predict xerostomia in patients with head and neck cancer. Adv Radiat Oncol.

[51] Sheikh K, Lee SH, Cheng Z, Lakshminarayanan P, Peng L, Han P, McNutt TR, Quon H, Lee J (2019). Predicting acute radiation induced xerostomia in head and neck cancer using MR and CT radiomics of parotid and submandibular glands. Radiat Oncol.

[52] Papachristou N, Puschmann D, Barnaghi P, Cooper B, Hu X, Maguire R, Apostolidis K, Conley YP, Hammer M, Katsaragakis S, Kober KM, Levine JD, McCann L, Patiraki E, Furlong EP, Fox PA, Paul SM, Ream E, Wright F, Miaskowski C (2018). Learning from data to predict future symptoms of oncology patients. PLoS One.

[53] Zhang Z, Zhu Y, Zhang L, Wang Z, Wan H (2018). Prediction model of critical weight loss in cancer patients during particle therapy. Jpn J Clin Oncol.

[54] Olling K, Nyeng DW, Wee L (2018). Predicting acute odynophagia during lung cancer radiotherapy using observations derived from patient-centred nursing care. Tech Innov Patient Support Radiat Oncol.

[55] Gabryś HS, Buettner F, Sterzing F, Hauswald H, Bangert M (2018). Design and selection of machine learning methods using radiomics and dosiomics for normal tissue complication probability modeling of xerostomia. Front Oncol.

[56] Lötsch J, Sipilä R, Tasmuth T, Kringel D, Estlander A, Meretoja T, Kalso E, Ultsch A (2018). Machine-learning-derived classifier predicts absence of persistent pain after breast cancer surgery with high accuracy. Breast Cancer Res Treat.

[57] Abdollahi H, Mostafaei S, Cheraghi S, Shiri I, Rabi Mahdavi S, Kazemnejad A (2018). Cochlea CT radiomics predicts chemoradiotherapy induced sensorineural hearing loss in head and neck cancer patients: a machine learning and multi-variable modelling study. Phys Med.

[58] van Dijk LV, Thor M, Steenbakkers RJ, Apte A, Zhai T, Borra R, Noordzij W, Estilo C, Lee N, Langendijk JA, Deasy JO, Sijtsema NM (2018). Parotid gland fat related magnetic resonance image biomarkers improve prediction of late radiation-induced xerostomia. Radiother Oncol.

[59] Cvetković J (2017). Breast cancer patients' depression prediction by machine learning approach. Cancer Invest.

[60] van Dijk LV, Brouwer CL, van der Schaaf A, Burgerhof JG, Beukinga RJ, Langendijk JA, Sijtsema NM, Steenbakkers RJ (2017). CT image biomarkers to improve patient-specific prediction of radiation-induced xerostomia and sticky saliva. Radiother Oncol.

[61] Jensen SB, Vissink A, Limesand KH, Reyland ME (2019). Salivary gland hypofunction and xerostomia in head and neck radiation patients. J Natl Cancer Inst Monogr.

[62] Fervaha G, Izard JP, Tripp DA, Rajan S, Leong DP, Siemens DR (2019). Depression and prostate cancer: a focused review for the clinician. Urol Oncol.

[63] Slovacek L, Slovackova B, Slanska I, Petera J, Priester P, Filip S, Kopecky J (2009). Depression symptoms and health-related quality of life among patients with metastatic breast cancer in programme of palliative cancer care. Neoplasma.

[64] Webber K, Davies AN, Leach C, Waghorn M (2023). Symptom prevalence and severity in palliative cancer medicine. BMJ Support Palliat Care.

[65] Zomkowski K, Cruz de Souza B, Pinheiro da Silva F, Moreira GM, de Souza Cunha N, Sperandio FF (2018). Physical symptoms and working performance in female breast cancer survivors: a systematic review. Disabil Rehabil.

[66] Bean HR, Diggens J, Ftanou M, Weihs KL, Stanton AL, Wiley JF (2021). Insomnia and fatigue symptom trajectories in breast cancer: a longitudinal cohort study. Behav Sleep Med.

[67] Pozzar RA, Hammer MJ, Cooper BA, Kober KM, Chen L, Paul SM, Conley YP, Levine JD, Miaskowski C (2021). Symptom clusters in patients with gynecologic cancer receiving chemotherapy. Oncol Nurs Forum.

[68] Harris CS, Kober KM, Conley YP, Dhruva AA, Hammer MJ, Miaskowski CA (2022). Symptom clusters in patients receiving chemotherapy: a systematic review. BMJ Support Palliat Care.

[69] Vabalas A, Gowen E, Poliakoff E, Casson AJ (2019). Machine learning algorithm validation with a limited sample size. PLoS One.

[70] Balki I, Amirabadi A, Levman J, Martel AL, Emersic Z, Meden B, Garcia-Pedrero A, Ramirez SC, Kong D, Moody AR, Tyrrell PN (2019). Sample-Size determination methodologies for machine learning in medical imaging research: a systematic review. Can Assoc Radiol J.

[71] Rahmani AM, Yousefpoor E, Yousefpoor MS, Mehmood Z, Haider A, Hosseinzadeh M, Ali Naqvi R (2021). Machine learning (ML) in medicine: review, applications, and challenges. Mathematics.

[72] Cabitza F, Campagner A, Soares F, García de Guadiana-Romualdo L, Challa F, Sulejmani A, Seghezzi M, Carobene A (2021). The importance of being external methodological insights for the external validation of machine learning models in medicine. Comput Methods Programs Biomed.

