# Supplementary material for: Machine Learning Approaches to Predict Symptoms in People With Cancer: Systematic Review
Source: JMIR Cancer. 2024 Mar 19;10:e52322. doi: 10.2196/52322 (PMC10988375; doi:10.2196/52322)
Supplement: Multimedia Appendix 1 [file cancer_v10i1e52322_app1.docx]

Research Terms

In our research, we have employed a detailed and comprehensive approach using both MeSH terms, CINAHL headings, Emtree terms, and specific keywords/phrases. This multi-faceted strategy ensures a thorough and inclusive representation of cancer and its related symptoms, aligning with the aims of our study on machine learning and cancer symptom prediction.

Conceptual Definition: Cancer, in our study, is conceptualized broadly to encompass a range of neoplastic conditions and symptoms associated with cancer. This concept extends beyond the mere presence of neoplastic cells to include the various physiological, psychological, and neurological symptoms that cancer patients experience.

Operational Definition:

Search Terms Utilized: Our operational definition of cancer is mirrored in the diversity and specificity of our search terms. We have used a combination of MeSH terms, CINAHL headings, Emtree terms, and specific keywords/phrases to capture the multifaceted nature of cancer and its symptoms.

MeSH Terms: These include 'Neoplasms' coupled with various symptoms like 'Cancer pain', 'Fatigue', 'Neuralgia', etc., providing a comprehensive capture of cancer-related literature in medical databases.

CINAHL Headings: Like MeSH, but tailored for the CINAHL database, we have used terms like 'Cancer pain', 'Cancer fatigue', and others, ensuring a broad yet specific retrieval of relevant literature.

Emtree Terms: Employed for Embase database searches, these terms like 'Neoplasms', 'Cancer pain', and 'Cancer fatigue', help in identifying pertinent studies related to cancer and its myriad symptoms.

Keywords/Phrases: Beyond standard indexing terms, we have also included a variety of keywords and phrases such as 'Cancer OR neoplasm', 'Fatigue', 'Loss of energy', 'Anxiety', 'Depression', etc., to encompass a wide array of cancer-related symptoms and conditions. This approach ensures that we capture studies that might use non-standard terminology or newly emerging terms not yet indexed in the databases.

Our methodology, by integrating these diverse and exhaustive search terms, ensures that we operationalize the concept of cancer in a manner that is both inclusive and reflective of the current scientific understanding of the disease and its impact on patients. This approach is crucial for a study that aims to use machine learning for predicting cancer symptoms, as it requires a nuanced understanding of the various ways cancer, and its symptoms are described and reported in the medical literature.

Research terms:

| Concept 1: machine learning | Concept 2: cancer symptoms |
| --- | --- |
| MeSH  Machine learning (includes deep learning, supervised and unsupervised machine learning)  Data mining  Neural networks, computer  Decision tree | MeSH  Cancer pain  Neoplasms  AND  Signs and symptoms  Pain  Symptom assessment  Fatigue  Neuralgia  Anxiety  Depression  Edema  Xerostomia  Dyssomnias  Constipation  Dyspnea  Nausea  Vomiting  Cachexia  Headache  hyperesthesia  Pruritus  Memory disorders  Anorexia |
| CINAHL headings  Machine learning (includes deep learning, support vector machine)  Data mining  Neural networks, computer  Decision tree | CINAHL headings  Cancer pain  Cancer fatigue  Neoplasms  AND  Signs and symptoms  Pain  Fatigue  Neuralgia  Anxiety  Depression  Edema  Xerostomia  Dyssomnias  Constipation  Dyspnea  Nausea  Vomiting  Cachexia  Headache  hyperesthesia  Pruritus  Memory disorders  Anorexia |
| Emtree terms  Machine Learing  Data mining  Decision tree  Artificial neural networks | Emtree terms  Cancer pain  Cancer fatigue  Neoplasms  AND  Pain  Fatigue  Neuralgia  Anxiety  Depression  Edema  Xerostomia  Sleep disorders  Constipation  Dyspnea  Nausea  Vomiting  Cachexia  Headache  hyperesthesia  Pruritus  Memory disorders  Loss of appetite |
| Keywords/phrases  Computer neural network  Computational neural network  Deep learning  Machine learning  Hierarchical learning  Support vector machine  Support vector network (s)  Logistic regression  Logistic regression(s)  Artificial Neural Network  Artificial Neural Network(s)  K-Nearest Neighbor  K-Nearest Neighbor(s)  random forest  decision tree  decision tree(s)  Extreme Gradient Boosting  Naive Bayes  K-means  convolutional neural network  convolutional neural network(s)  recurrent neural network  recurrent neural network(s) | Keywords/phrases  Cancer symptoms  Cancer OR neoplasm  Fatigue  Loss of energy  Neuralgia  Anxiety  Anxious  Depression  Depressed  Swelling  Edema  Dry Mouth  Xerostomia  Disturbed Sleep  Insomnia  Sleep problems  Dyssomnia  Constipation  Shortness of breath  Dyspnea  breathlessness  Nausea  Vomiting  pain  Decreased appetite  Appetite loss  Loss of appetite  Reduced appetite  Headache(s)  Head pain  Hyperesthesia  Numbness  paresthesia  Pruritus  Itching  Impaired memory  Memory loss |

Explain why the use of Boolean expressions adds rigor to the methodology?

The use of Boolean expressions in our systematic review is fundamental for defining the search scope with precision. These expressions, through specific combinations of keywords and phrases, enable us to accurately target the most relevant literature. For instance, the operator 'AND' is used to narrow down the search by combining terms, ensuring that only studies containing all the specified terms are retrieved.

Conversely, the operator 'OR' broadens the search to encompass any of the terms. This is particularly useful for including synonyms or related terms, thereby ensuring comprehensive coverage. For example, "neoplasm OR cancer" captures literature using either term, acknowledging the variability in terminology across studies.

In enhancing inclusivity and exclusivity, Boolean expressions enable researchers to include all relevant studies, thereby maximizing sensitivity, while simultaneously excluding irrelevant ones to increase specificity. This dual function is essential for maintaining the quality and relevance of the systematic review. By carefully selecting and combining terms using Boolean logic, researchers can strike a balance between capturing as many relevant studies as possible (sensitivity) and avoiding the inclusion of irrelevant studies (specificity). Furthermore, tailoring the search to the research question is facilitated by Boolean expressions, ensuring that the literature review is directly relevant to the aims and objectives of the study. In summary, Boolean expressions contribute significantly to the methodology's rigor by enabling a targeted and comprehensive search strategy. This strategy ensures that the literature review is both thorough and relevant, enhancing the overall quality and credibility of the research.

The impact on results quality is notable. Boolean expressions contribute to the reliability and comprehensiveness of a literature review. By allowing for precise and nuanced searches, they ensure that the literature search is exhaustive and captures all relevant studies. This thoroughness is critical for the integrity of a systematic review, as it minimizes the risk of omitting key studies that could influence the review's conclusions. Boolean expressions are instrumental in filtering the vast amount of available literature to identify studies most pertinent to the research question. For example, combining broad terms with more specific ones using 'AND' helps in zeroing in on studies that are not just about cancer in general, but specifically address the research interest, such as cancer-related nausea. Regarding the reduction of bias, the use of Boolean expressions helps mitigate selection bias by defining clear, objective, and reproducible criteria for including or excluding studies. A well-defined Boolean search strategy is transparent and can be replicated by other researchers, lending credibility to the review process. This consistency is crucial in ensuring that the search results are not biased by the subjective preferences of the researcher. Furthermore, by using a combination of broad and narrow terms, and including relevant synonyms and related terms, Boolean expressions ensure a balanced search. This balance is key to avoiding an overly narrow focus that might miss important literature or an overly broad scope that pulls in too much irrelevant information.

In conclusion, Boolean expressions enhance the quality of results in a systematic review by ensuring a comprehensive, targeted, and unbiased search. They are fundamental in creating a search strategy that is both exhaustive and specific to the research question, thereby enhancing the reliability of the review and reducing the risk of selection bias.

|  | **Multimedia Appendix 1** Databases search strategy  PubMed 1993 to Aug 11, 2023.  ((("cancer"[Title/Abstract] OR "neoplasm"[Title/Abstract] OR "Neoplasms"[Title/Abstract]) AND ("Fatigue"[Title/Abstract] OR "loss of energy"[Title/Abstract] OR "Neuralgia"[Title/Abstract] OR "Anxiety"[Title/Abstract] OR "Anxious"[Title/Abstract] OR "Depression"[Title/Abstract] OR "Depressed"[Title/Abstract] OR "Swelling"[Title/Abstract] OR "Edema"[Title/Abstract] OR "dry mouth"[Title/Abstract] OR "Xerostomia"[Title/Abstract] OR "disturbed sleep"[Title/Abstract] OR "Insomnia"[Title/Abstract] OR "sleep disorders"[Title/Abstract] OR "Dyssomnia"[Title/Abstract] OR "Constipation"[Title/Abstract] OR "shortness of breath"[Title/Abstract] OR "Dyspnea"[Title/Abstract] OR "breathlessness"[Title/Abstract] OR "Nausea"[Title/Abstract] OR "Vomiting"[Title/Abstract] OR "Pain"[Title/Abstract] OR "decreased appetite"[Title/Abstract] OR "appetite loss"[Title/Abstract] OR "loss of appetite"[Title/Abstract] OR "reduced appetite"[Title/Abstract] OR "Headache"[Title/Abstract] OR "Headaches"[Title/Abstract] OR "head pain"[Title/Abstract] OR "Hyperesthesia"[Title/Abstract] OR "Numbness"[Title/Abstract] OR "paresthesia"[Title/Abstract] OR "Pruritus"[Title/Abstract] OR "Itching"[Title/Abstract] OR "impaired memory"[Title/Abstract] OR "memory loss"[Title/Abstract])) OR ("cancer symptom"[Title/Abstract] OR "cancer symptoms"[Title/Abstract]) OR ("Cancer Pain"[MeSH Terms] OR (("Signs and Symptoms"[MeSH Terms] OR "Pain"[MeSH Terms] OR "Symptom Assessment"[MeSH Terms] OR "Fatigue"[MeSH Terms] OR "Anxiety"[MeSH Terms] OR "Depression"[MeSH Terms] OR "Edema"[MeSH Terms] OR "Xerostomia"[MeSH Terms] OR "Dyssomnias"[MeSH Terms] OR "Constipation"[MeSH Terms] OR "Dyspnea"[MeSH Terms] OR "Nausea"[MeSH Terms] OR "Vomiting"[MeSH Terms] OR "Cachexia"[MeSH Terms] OR "Headache"[MeSH Terms] OR "Hypesthesia"[MeSH Terms] OR "Pruritus"[MeSH Terms] OR "Memory Disorders"[MeSH Terms]) AND "Neoplasms"[MeSH Terms]))) AND ("computer neural network"[Title/Abstract] OR "computational neural network"[Title/Abstract] OR "deep learning"[Title/Abstract] OR "Machine Learning"[Title/Abstract] OR "hierarchical learning"[Title/Abstract] OR "support vector machine"[Title/Abstract] OR "support vector network"[Title/Abstract] OR "support vector networks"[Title/Abstract] OR "Logistic regressions"[Title/Abstract] OR "Artificial Neural Network"[Title/Abstract] OR "Artificial Neural Networks"[Title/Abstract] OR "K-Nearest Neighbor"[Title/Abstract] OR "K-Nearest Neighbors"[Title/Abstract] OR "random forest"[Title/Abstract] OR "decision tree"[Title/Abstract] OR "decision trees"[Title/Abstract] OR "Extreme Gradient Boosting"[Title/Abstract] OR "Naïve Bayes"[Title/Abstract] OR "K-means"[Title/Abstract] OR "convolutional neural network"[Title/Abstract] OR "convolutional neural networks"[Title/Abstract] OR "recurrent neural network"[Title/Abstract] OR "recurrent neural networks"[Title/Abstract] OR ("Machine Learning"[MeSH Terms] OR "neural networks, computer"[MeSH Terms] OR "Data Mining"[MeSH Terms]))  Embase 1990 to Aug 11, 2023.  S1:  'neural networks' OR 'data mining' OR 'support vector machine' OR 'k nearest neighbor' OR 'random forest' OR 'decision tree' OR 'extreme gradient boosting' OR 'naïve bayes classifier' OR 'k means clustering' OR 'convolutional neural network' OR 'recurrent neural network' OR 'artificial neural network' OR 'deep learning' OR 'deep learning algorithm' OR 'deep learning model' OR 'machine learning' OR 'logistic regressions'  S2:  'symptoms' OR 'nausea and vomiting' OR 'cachexia' OR 'memory disorder' OR 'anorexia' OR 'fatigue' OR 'loss of energy' OR 'neuralgia' OR 'anxiety' OR anxious OR 'depression' OR 'swelling' OR 'edema' OR 'dry mouth' OR 'xerostomia' OR 'disturbed sleep' OR 'insomnia' OR 'sleep disorder' OR dyssomnia OR 'constipation' OR 'shortness of breath' OR 'dyspnea' OR breathlessness OR 'nausea' OR 'vomiting' OR 'pain' OR 'decreased appetite' OR 'loss of appetite' OR 'appetite loss' OR 'reduced appetite' OR 'headache' OR 'head pain' OR 'hyperesthesia' OR numbness OR 'paresthesia' OR 'pruritus' OR itching OR 'impaired memory' OR 'memory loss' OR 'amnesia'  S3:  'Neoplasm'  S4:  'cancer pain' OR 'cancer fatigue'  S5:  #3 OR #4  S6:  #2 AND #5  S7:  # 1 AND #6  CINAL 1992 to Aug 11, 2023.  S1: (MH "Machine Learning+") OR (MH "Neural Networks (Computer)") OR (MH "Data Mining+") OR (MH "Decision Trees+")  S2: (MH "Cancer Pain") OR (MH "Cancer Fatigue")  S3: (MH "Neoplasms+")  S4: (MH "Signs and Symptoms+") OR (MH "Pain+") OR (MH "Fatigue+") OR (MH "Neuralgia+") OR (MH "Anxiety+") OR (MH "Depression+") OR (MH "Edema+") OR (MH "Xerostomia+") OR (MH "Dyssomnias+") OR (MH "Constipation+") OR (MH "Dyspnea+") OR (MH "Nausea") OR (MH "Vomiting+") OR (MH "Cachexia") OR (MH "Headache+") OR (MH "Hyperesthesia") OR (MH "Pruritus") OR (MH "Memory Disorders+")  S5: S3 AND S4  S6: S2 OR S5  S7: S1 AND S6  S8: (MH "Logistic Regression+")  S9: S1 AND S8  S10: "Computer neural network” OR “Computational neural network” OR “Deep learning” OR “Machine learning” OR “Hierarchical learning”  S11: “Support vector machine” OR “Support vector network*” OR “Artificial Neural Network*” OR “K-Nearest Neighbor*” OR “random forest” OR “decision tree*” OR “Extreme Gradient Boosting” OR “Naïve Bayes” OR “K-means” OR “convolutional neural network*” OR “recurrent neural network*” OR “logistic regressions”  S13: S1 OR S10 OR S11  S14: "cancer symptom*"  S15: (Cancer* OR neoplasm*) AND (Fatigue OR “Loss of energy” OR Neuralgia OR Anxiety OR Anxious OR Depress* OR Swelling OR Edema OR “Dry Mouth” OR Xerostomia OR “Disturbed Sleep” OR Insomnia OR “Sleep disorder*” OR Dyssomnia* OR Constipation OR “Shortness of breath” OR Dyspnea OR breathlessness OR Nausea OR Vomiting OR pain OR “Decreased appetite” OR “Appetite loss” OR “Loss of appetite” OR “Reduced appetite” OR Headache* OR “Head pain” OR Hyperesthesia OR Numbness OR paresthesia OR Pruritus OR Itching OR “Impaired memory” OR “Memory loss”)  S16: S6 OR S14 OR S15  S17: S13 AND S16 |
| --- | --- |
