# Supplementary figures and images for: Machine Learning Approaches to Predict Symptoms in People With Cancer: Systematic Review
Source: JMIR Cancer. 2024 Mar 19;10:e52322. doi: 10.2196/52322 (PMC10988375; doi:10.2196/52322)

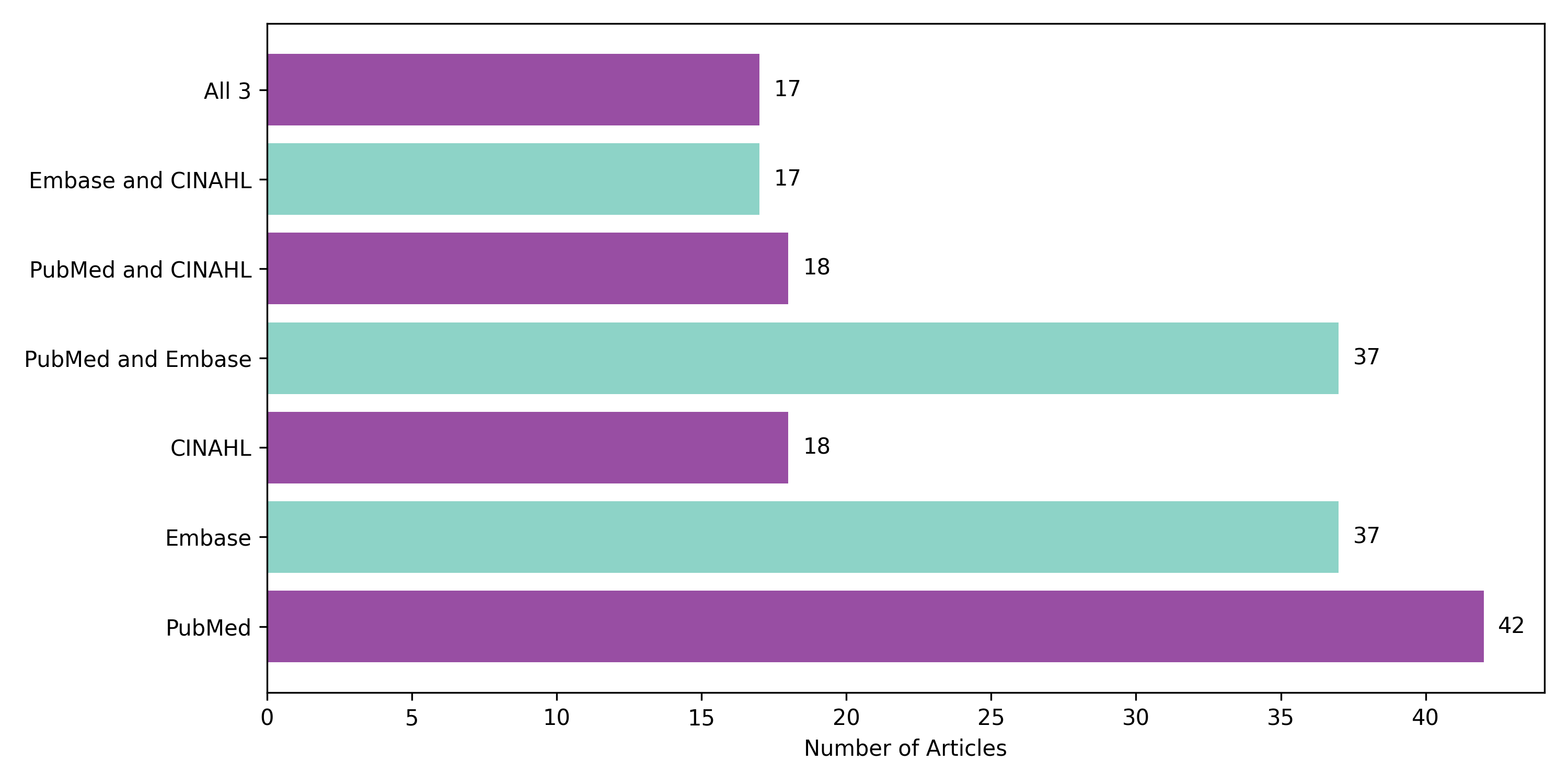

Supplement: Multimedia Appendix 3 [file cancer_v10i1e52322_app3.png]

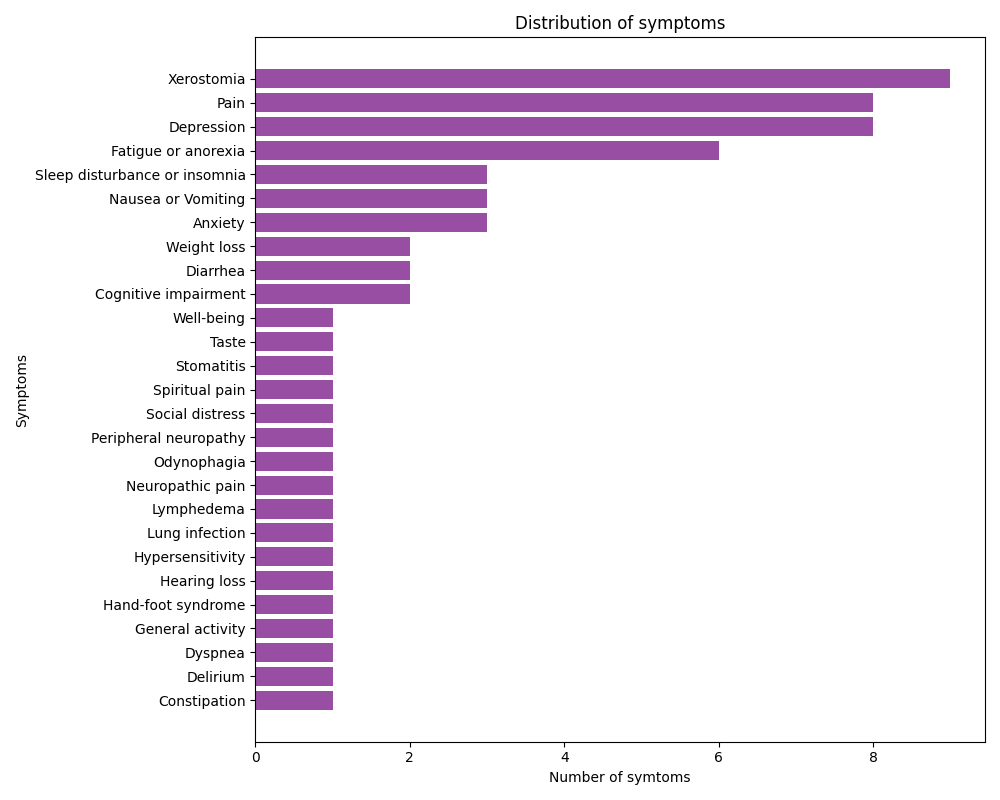

Supplement: Multimedia Appendix 5 [file cancer_v10i1e52322_app5.png]

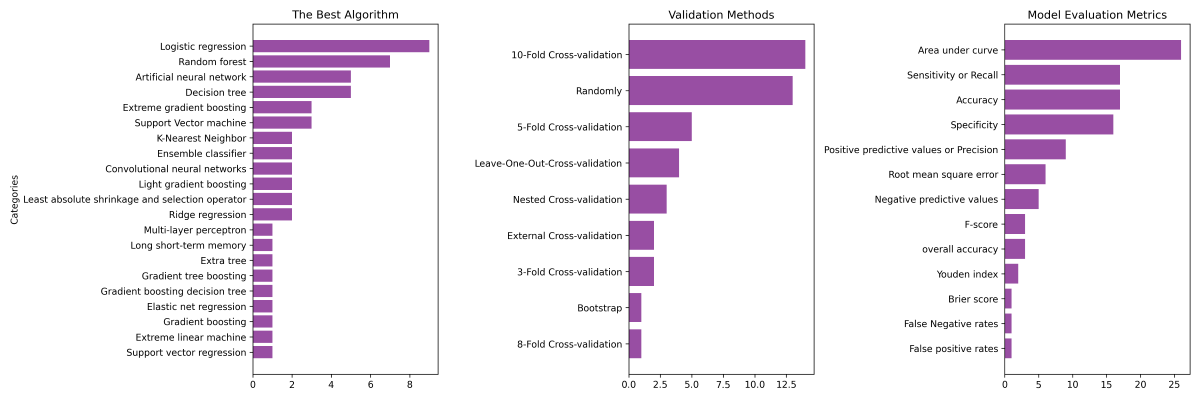

Supplement: Multimedia Appendix 6 [file cancer_v10i1e52322_app6.png]
