# Supplementary material for: Machine Learning Approaches to Predict Symptoms in People With Cancer: Systematic Review
Source: JMIR Cancer. 2024 Mar 19;10:e52322. doi: 10.2196/52322 (PMC10988375; doi:10.2196/52322)
Supplement: Multimedia Appendix 4 [file cancer_v10i1e52322_app4.docx]

Table 2. The Summary of Included Studies (N=42)

| Characteristics | Categories | Number (Percentage) ^a^ |
| --- | --- | --- |
| Place of Research |  |  |
|  | North America | 18(43) |
|  | Asia | 16(38) |
|  | Europe | 8(19) |
| Number of Involved Centers |  |  |
|  | Single center | 23(55) |
|  | Multiple centers | 19(45) |
| Sample Size |  |  |
|  | <100 | 8(19) |
|  | 100~1000 | 27(64) |
|  | >1000 | 7(17) |
| Type of Features |  |  |
|  | Clinical features | 28(67) |
|  | Clinical + Image features | 14(33) |
| Cancer Site |  |  |
|  | Head and Neck | 9(22) |
|  | Breast | 8(19) |
|  | Lung | 3(7) |
|  | Colorectal | 1(2) |
|  | Oropharyngeal | 1(2) |
|  | Pancreatic | 1(2) |
|  | Gastrointestinal | 1(2) |
|  | Non-Hodgkin’s lymphoma | 1(2) |
|  | Not mentioned | 17(40) |
| Symptoms |  |  |
|  | Xerostomia | 9(14) |
|  | Pain | 8(13) |
|  | Depression | 8(13) |
|  | Fatigue/anorexia | 6(10) |
|  | Anxiety | 3(5) |
|  | Sleep disturbance/Insomnia | 3(5) |
|  | Nausea/Vomiting | 3(5) |
|  | Weight Loss | 2(3) |
|  | Cognitive Impairment | 2(3) |
|  | Diarrhea | 2(3) |
|  | Hypersensitivity | 1(2) |
|  | Stomatitis | 1(2) |
|  | Hand-foot Syndrome | 1(2) |
|  | Peripheral Neuropathy | 1(2) |
|  | Constipation | 1(2) |
|  | Delirium | 1(2) |
|  | Lung Infection | 1(2) |
|  | Well-being | 1(2) |
|  | Lymphedema | 1(2) |
|  | Taste | 1(2) |
|  | General Activity | 1(2) |
|  | Neuropathic Pain | 1(2) |
|  | Odynophagia | 1(2) |
|  | Hearing Loss | 1(2) |
|  | Social Distress | 1(2) |
|  | Dyspnea | 1(2) |
|  | Spiritual Pain | 1(2) |
| Type of Study |  |  |
|  | Restorative Observational | 18(43) |
|  | Prospective Observational | 5(12) |
|  | Cross-sectional | 15(38) |
|  | Longitudinal | 4(10) |
| Treatment |  |  |
|  | Chemotherapy | 9(21) |
|  | Radiotherapy | 9(21) |
|  | Surgery | 4(10) |
|  | Post-treatment survivors | 2(5) |
|  | Palliative care setting | 1(2) |
|  | Not mention | 17(40) |
| Number of Machine Learning Algorithms |  |  |
|  | 1 | 7(17) |
|  | >1 | 35(83) |
| Category of Machine Learning Algorithms |  |  |
|  | Supervised Machine Learning | 39(93) |
|  | Else (Unsupervised Machine Learning, Deep Learning, Ensemble) | 3(1,3,3) |
| The Best Algorithm |  |  |
|  | Logistic regression (LR) | 9(17) |
|  | Random Forest (RF) | 7(13) |
|  | Artificial Neural Network (ANN) | 5(9) |
|  | Decision Tree (DT) | 5(9) |
|  | Extreme Gradient Boosting (XGB) | 3(6) |
|  | SVM | 3(6) |
|  | Ensemble Classifier | 2(4) |
|  | Convolutional Neural Networks (CNN) | 2(4) |
|  | Light Gradient Boosting (Light GBM) | 2(4) |
|  | K-Nearest Neighbor (KNN) | 2(4) |
|  | Least Absolute Shrinkage and Selection Operator (LASSO) | 2(4) |
|  | Ridge Regression (RR) | 2(4) |
|  | Long Short-term Memory (LSTM) | 1(2) |
|  | Extra Tree (ET) | 1(2) |
|  | Gradient Tree Boosting (GTB) | 1(2) |
|  | Gradient Boosting Decision Tree (GBDT) | 1(2) |
|  | Elastic Net Regression (EN) | 1(2) |
|  | Gradient Boosting (GB) | 1(2) |
|  | Elastic Net Regression (ENR) | 1(2) |
|  | Extreme Linear Machine (ELM) | 1(2) |
|  | Multi-Layer Perceptron (MLP) | 1(2) |
|  | Support Vector Regression (SVR) | 1(2) |
| Validation Methods |  |  |
|  | 3-Fold Cross Validation | 2(4) |
|  | 5-Fold Cross Validation | 5(11) |
|  | 8-Fold Cross Validation | 1(2) |
|  | 10-Fold Cross Validation | 14(31) |
|  | Leave-One-Out-Cross-Validation | 4(9) |
|  | Nested CV | 3(7) |
|  | External CV | 2(4) |
|  | Bootstrap | 1(2) |
|  | Randomly | 13(29) |
| Model Evaluation Metrics |  |  |
|  | Area Under Curve | 26(24) |
|  | Accuracy | 17(16) |
|  | Sensitivity /Recall | 17(16) |
|  | Specificity | 16(15) |
|  | Positive Predictive Values /Precision | 9(8) |
|  | Root Mean Square Error | 6(6) |
|  | Negative Predictive Values | 5(5) |
|  | overall Accuracy | 3(3) |
|  | F1 score | 3(3) |
|  | Youden’s index | 2(2) |
|  | False Positive Rates | 1(1) |
|  | False Negative Rates | 1(1) |
|  | Brier Score | 1(1) |
| Feature Selection |  |  |
|  | Yes | 30(71) |
|  | No | 12(29) |

a. Percentages are calculated based on the total number of studies (N=42).
